# Management of particular clinical situations in psoriatic arthritis: an expert’s recommendation document based on systematic literature review and extended Delphi process

**DOI:** 10.1007/s00296-021-04877-5

**Published:** 2021-05-02

**Authors:** Rosario García-Vicuña, Noemí Garrido, Susana Gómez, Beatriz Joven, Rubén Queiro, Julio Ramírez, Francisco Rebollo, Estíbaliz Loza, Agustí Sellas

**Affiliations:** 1 Servicio de Reumatología, Hospital Universitario La Princesa, IIS-IP, Calle de Diego de León 62, 28006 Madrid, Spain; 2 Servicio de Reumatología, Hospital Universitario Virgen del Rocío, Sevilla, Spain; 3 Pfizer Medical Department, Alcobendas, Madrid, Spain; 4 Servicio de Reumatología, Hospital Universitario 12 de Octubre, Madrid, Spain; 5 Sección de Reumatología, Hospital Universitario Central de Asturias, Oviedo, Spain; 6 Servicio de Reumatología, Hospital Clínic, Barcelona, Spain; 7 Instituto de Salud Musculoesquelética, Madrid, Spain; 8 Servicio de Reumatología, Hospital Universitari Arnau de Vilanova, Lleida, Spain

**Keywords:** Psoriatic arthritis, Manifestations, Disease-modifying antirheumatic drugs, Recommendations, Systematic literature review

## Abstract

To establish practical recommendations for the management of patients with psoriatic arthritis (PsA) with particular clinical situations that might lead to doubts in the pharmacological decision-making. A group of six expert rheumatologists on PsA identified particular clinical situations in PsA. Then, a systematic literature review (SLR) was performed to analyse the efficacy and safety of csDMARDs, b/tsDMARDs in PsA. In a nominal group meeting, the results of the SLR were discussed and a set of recommendations were proposed for a Delphi process. A total of 65 rheumatologists were invited to participate in the Delphi. Agreement was defined if ≥ 70% of the participants voted ≥ 7 (from 1, totally disagree to 10, totally agree). For each recommendation, the level of evidence and grade of recommendation was established based on the Oxford Evidence-Based Medicine categorisation. Particular clinical situations included monoarthritis, axial disease, or non-musculoskeletal manifestations. The SLR finally comprised 131 articles. A total of 16 recommendations were generated, all but 1 reached consensus. According to them, it is crucial to carefully analyse the impact of individual manifestations on patients (disability, quality of life, etc.), but also to recognise the impact of each drug singularities on selected clinical phenotypes to adopt the most appropriate treatment strategy. Early diagnosis and treatment to target approach, along with a close risk management, is also necessary. These recommendations are intended to complement gaps in national and international guidelines by helping health professionals address and manage particular clinical situations in PsA.

**Supplementary Information:**

The online version contains supplementary material available at 10.1007/s00296-021-04877-5.

## Introduction

Psoriatic arthritis (PsA) is a heterogeneous, potentially severe and complex disease [1]. It comprises multiple disease manifestations, including peripheral arthritis, enthesitis, dactylitis, spondylitis, skin and nail psoriasis, but also other manifestations like inflammatory bowel disease or uveitis [2]. PsA is also an immune-mediated systemic inflammatory disease associated with cardiovascular disease or events, psychological disorders and other comorbidities. As a consequence, PsA is associated with a great impact on patients’ quality of life and an increased mortality risk [3].

On the other hand, different therapeutic agents are currently available for PsA patients [4]. Therapeutic options include conventional synthetic disease-modifying antirheumatic drugs DMARDs (csDMARDs), biologic DMARDs (bDMARDs) and targeted synthetic DMARDs (tsDMARDs) including the phosphodiesterase-4 (PDE4) inhibitor apremilast, or Janus kinases (JAKs) inhibitors like tofacitinib [5]. Along with new treatments, a treat-to-target (T2T) strategy has been recommended and implemented for PsA [6]. Although there are still several major areas of ongoing unmet needs in the care of patients with PsA, an improvement in clinical outcomes has been recognised in recent years suggesting that new treatments and strategies are effective [7, 8].

In this context, randomised controlled trials (RCTs) have been central to demonstrate the efficacy of these drugs in PsA. However, most of them have analysed outcomes in the short or medium term, using mainly placebo as comparator. Direct comparative research of different drugs, important for clinical practice, is rather scarce in PsA. Besides, considering the strict inclusion criteria of RCTs or extrapolations from RA studies, study populations might not be representative of ‘real-life’ patients and, thus, results lack generalisability [9]. This issue is of key relevance in PsA taking into account the clinical heterogeneity of the disease and that comorbidities (a frequent exclusion criteria) are often present. Similarly, RCTs design and primary end-points are mainly focused on arthritis, being PsA a multifaceted domain disease.

On the other hand, consensus documents and clinical guidelines aim to analyse the best evidence to provide some guidance in treatment decision-making, even in situations where evidence is insufficient or even absent [4, 10–13]. They are usually focused on the most relevant patient’s profiles. However, in daily practice physicians have to treat patients with particular clinical situations that are not specifically covered in these documents.

Considering all of the exposed above, and to complement and try to approach some gaps in national and international guidelines, we set the following objectives (1) to define particular clinical situations, (2) to collect and analyse the best available evidence by conducting a systematic literature review (SLR), and (3) to generate practical recommendations to guide physicians in the management of multidomain complex PsA patients.

## Methods

The nominal group and *Delphi* techniques were used. The document was generated via distribution of tasks, with the help of a systematic literature review (SLR), and under the supervision of a methodologist.

### First nominal meeting group

A steering group consisting of 6 experts on PsA was established. The criteria for the selection of experts were: specialised rheumatologists in PsA, clinical experience ≥ 8 years and / or ≥ 5 publications on PsA, and members of the Spanish Society of Rheumatology (SER) and related working groups on PsA.

In the first *on-line* nominal meeting group, the steering group established the objectives, scope, users, definitions, and document contents. Besides, particular clinical situations in PsA were also identified. With all this information, the experts defined the inclusion and exclusion criteria for a subsequent SLR.

### Systematic literature review

A SLR was conducted in accordance with the Preferred Reporting Items for Systematic Reviews and Meta-Analyses (PRISMA) guidelines, which followed the Good Clinical Practice regulations. A protocol pertaining to the review was then designed. The research question “Which is the efficacy and safety of csDMARDs, bDMARDs and tsDMARDs in particular clinical situations in PsA” was translated to a PICO question. Studies were identified using sensitive search strategies in the main medical databases. For this purpose, an expert librarian checked the search strategies (supplementary data). Disease- and treatment-related terms were used as search keywords, which employed a controlled vocabulary, specific MeSH headings, and additional keywords. The following bibliographic databases were screened: Medline (up to March 2020), Embase (up to March 2020), and Cochrane Library (up to March 2020). Retrieved references were managed in Endnote X5 (Thomson Reuters). The abstracts of the 2018 and 2019 annual scientific meetings of the American College of Rheumatology (ACR) and the European Alliance of Associations for Rheumatology (EULAR) were also examined, along with national and international consensus documents and guidelines [4, 10–13].

Studies retrieved using the search strategies were included if they met the following pre-established criteria. Patients had to be diagnosed with PsA, aged 18 or older, with particular clinical situations, and treated with bDMARDs like tumour necrosis factor (TNF) inhibitors, abatacept, interleukin (IL)-17 inhibitors, IL-12/23 inhibitors, and ts/DMARDs like JAKs inhibitor tofacitinib, and the PDE4 inhibitor apremilast. There was no restriction regarding the type of drug, dose route of administration, concomitant use of other drugs, or treatment duration. Different outcomes were considered, such as pain, radiographic progression or quality of life. Only SLRs and RCTs in English or Spanish were included.

The screening of studies, data collection, and analysis were performed independently by two reviewers (EL and TO). In the case of discrepancy between reviewers, a consensus was reached by including a third reviewer (LC). To grade their quality, we used the Jadad score [14] for RCTs. Evidence tables were then produced, and meta-analysis was only planned if enough homogeneity (clinical and I^2^ ≤ 20%) among the included studies was observed.

### Second nominal meeting group

The steering group participated in a second on-line meeting group. Prior to the meeting, the results of the SLR were distributed. The experts, following the results of the SLR but also based on their experience, formulated recommendations for the management of particular clinical situations in PsA.

### Delphi

Recommendations were subsequently submitted to on-line Delphi voting (up to 3 rounds). Delphi was extended to a group of 65 rheumatologists (including the steering group). The participants voted each recommendation on a scale from 1 to 10 (1 = totally disagree, to 10 = totally agree). Agreement was defined if at least 70% of participants voted ≥ 7. Recommendations with a level of agreement (LA) inferior to 70% were analysed and, if appropriate, re-edited and voted in a second round.

### Final consensus document

After the Delphi, and along with the results of the SLR, the final document was written. The experts agreed to focus on the approval dose of tofacitinib 5 mg bid. The methodologist assisted in assigning each recommendation, a level of evidence (LE), and grade of recommendation (GR), according to the Center for Evidence-Based Medicine of Oxford [15]. The document circulated among the experts for final assessment and comments.

## Results

**Fig. 1:**
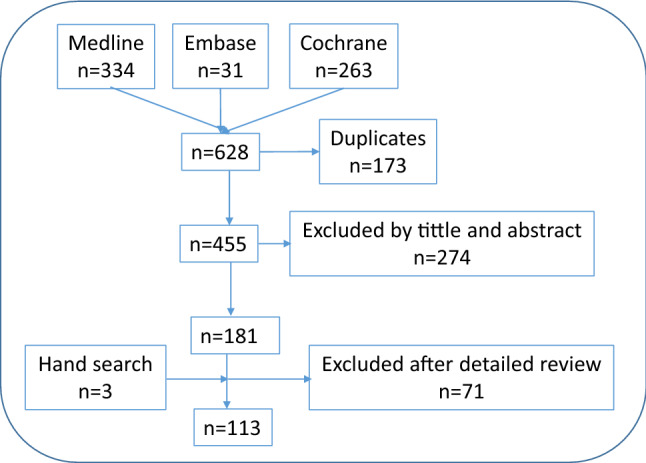
Studies flow chart

Several particular clinical situations were identified, like the presence of axial disease or dactylitis (Table 1). The SLR (designed to provide evidence for these specific patients) retrieved 181 articles of which 131 were finally included (Fig. 1). Most of them were articles of good quality. Based on their results and the experts experience and opinion, a total of 16 recommendations to guide treatment decisions in particular clinical situations. All but one reached consensus (see Table 2). The Delphi response rate was 46%.

**Table 1:** Particular clinical situations identified in the project

#	Particular clinical situations
1	Articular disease (mono and oligoarthritis)
2	Axial disease
3	Enthesitis
4	Dactylitis
5	Skin and nail disease
6	Non-musculoskeletal manifestations and comorbidities
7	Early PsA Risk management
8	Erosive disease
9	Mono- vs. combined therapy
10	Risk management

**Table 2:** Main results of the Delphi process

#	Recommendation	Mean	SD	Median	p25	p75	Min	Max	% ≥ 7*
1	Treatment and therapeutic strategy in PsA must be holistic, taking into account clinical findings and their impact on the patient's daily life	9.08	1.41	9	8.25	10	5	10	96%
2	In patients with monoarthritis, it is considered appropriate to start treatment with csDMARDs but also, prior to the start of csDMARDs, to consider intra-articular glucocorticoids injections or systemic short-term low-dose glucocorticoids	7.15	3.54	8	5.25	9	1	10	70%
3	It is recommended to consider a similar approach for oligoarthritis and polyarthritis	8.28	0.71	9	8	10	1	10	85%
4	It is recommended to select treatment according to predominant domain involvement (axial, peripheral or other) and its impact on patient	9.64	0.71	10	9	10	9	10	96%
5	In general, in patients with axial disease, it is recommended to be cautious with the use of NSAIDs (age of patient, presence of comorbidity, etc.)	8.96	1.41	9	8	10	6	10	92%
6	In patients with axial disease, oral or intramuscular short-term low-dose glucocorticoids (4–12 weeks) could be considered for axial symptoms	4.38	1.41	4	2	6	1	10	23%
7	In patients with enthesitis, a careful clinical history and physical examination should be performed to rule out other non-inflammatory conditions (plantar fasciitis, trochanteric bursitis, etc.), wide-spread pain syndromes or central sensitisation, especially in patients with multiple painful entheses	8.54	0.00	9	8	10	2	10	88%
8	In patients with enthesitis refractory to NSAIDs and/or local glucocorticoid injections, bDMARDs (except for abatacept), tofacitinib and apremilast are valid options in addition to reassess underlying inflammation with the use of imaging techniques such as ultrasound or MRI	8.69	0.00	9	8	10	4	10	92%
9	In patients with polyarthritis, concomitant dactylitis should be treated like polyarthritis	8.58	0.71	9	8	9	5	10	92%
10	In patients with dactylitis as predominant manifestation, before the start of csDMARDs or a treatment change, a more conservative treatment (1 or 2 local glucocorticoids injections) might be considered depending on the number of dactylitis and the impact on patient	7.88	0.00	8	8	9	1	10	88%
11	In patients with PsA and significant skin involvement, if a bDMARDs is considered, IL-17 or IL-12/23 inhibitors may be preferred	8.54	0.71	9	8.25	9	5	10	88%
12	Comorbidity should be systematically evaluated and managed in all patients with PsA	9.23	0.71	10	9	10	6	10	92%
13	In patients with PsA, an early and targeted treatment strategy like TICOPA is recommended	8.27	0.71	8.5	8	9	4	10	88%
14	In erosive PsA, early and tight treatment and monitoring is recommended	9.35	1.41	10	9	10	6	10	96%
15	In patients with PsA, the use of bDMARDs, or apremilast in monotherapy or in combination with csDMARDs, or tofacitinib in combination with csDMARDs, should be individualised	8.81	0.71	9	8	9.75	7	10	100%
16	Risk management recommendations for bDMARDs, tofacitinib and apremilast from regulatory agencies and scientific societies should be followed	9.23	2.12	9	9	10	7	10	100%

*SD* standard deviation, *Min* minumun, *max* maximum, *PsA* psoriatic arthritis, *csDMARDs* conventional synthetic disease-modifying antirheumatic drugs, *bDMARDs* disease-modifying antirheumatic drugs, *tsDMARDs*  targeted synthetic disease-modifying antirheumatic drugs, *NSAIDs* non-steroidal anti-inflammatory drugs, *IL*  interleukin

*Agreement was defined if at least 70% of participants voted ≥ 7.The participants voted each recommendation on a scale from 1 to 10 (1 = totally disagree, to 10 = totally agree)

Below, we summarise the findings of the SLR and the experts' opinion and attitude towards each particular clinical situations in PsA.

As recommended by international organisations and societies, initial treatment considerations in PsA should be based on discrete clinical manifestations, symptom severity and their impact on patients [4, 10–13]. The selection of initial treatment should also take into account comorbidities associated with PsA. And when making treatment decisions, it is important to consider that early and aggressive treatment may result in significant improvements in joint and skin symptoms, thus preventing permanent damage [16].

### Articular disease (mono and oligoarthritis)

#### Recommendations

1. Treatment and therapeutic strategy in PsA must be holistic, taking into account clinical findings and their impact on the patient's daily life (LE:5 / GR:D / LA:96%).
2. In patients with monoarthritis, it is considered appropriate to start treatment with csDMARDs but also, prior to the start of csDMARDs, to consider intra-articular glucocorticoids injections or systemic short-term low-dose glucocorticoids (LE:4 / GR:C / LA:70%).
3. It is recommended to consider a similar approach for oligoarthritis and polyarthritis (LE: 5 / GR:D /LA: 85%).

#### Evidence

The SLR found that csDMARDs are effective in PsA patients with peripheral arthritis, especially methotrexate (MTX) [17–21], but also other csDMARDs [22–29]. Although ciclosporin A has demonstrated efficacy [18, 30–32], it is usually not recommended probably due to safety issues and monitoring. bDMARDs including TNF inhibitors [30, 33–61], IL-17 inhibitors [62–77] and IL-12/23 inhibitors [78–82] have also depicted long-term efficacy. Similarly, tsDMARDs like tofacitinib [83, 84] or apremilast [85–90] have also demonstrated efficacy.

However, specific data on patients with monoarthritis or oligoarthritis in PsA are scarce [90, 91].

#### Expert´s comments and contributions

The experts agreed on a holistic approach in PsA. The impact of the disease on patients’ life should always be considered. Therefore, when making treatment decisions in PsA patients with articular manifestations, apart from assessing the pattern of joint involvement, type, number and severity of affected joint/s or the presence of other manifestations, the clinical impact on patient’s daily life should be evaluated, and patients treated accordingly. As an example, in clinical practice, we might face patients with knee monoarthritis presenting more limitations than others with hand oligoarthritis. The same way, more aggressive strategies might be considered in younger patients, or treatment changes according to patient’s comorbidities.

Monoarthritis is a common manifestation in early stages of the disease and there might be patients with no other relevant manifestations. In that case, the experts equally contemplate two scenarios: cDMARDs could be started, but either intra-articular glucocorticoids injections or short-term systemic low-dose glucocorticoids prior to the start of csDMARDs could be considered [92–94].

Oligoarthritis is also frequent in PsA. In these patients, intra-articular / systemic glucocorticoids might not be enough to control the disease or generate adverse events. Therefore, according to the experts, oligoarthritis should be considered as a polyarthritis.

### Axial disease

#### Recommendations

4. It is recommended to select treatment according to predominant domain involvement (axial, peripheral or other) and its impact on patient (LE:5 / GR:D /LA: 96%).
5. In general, in patients with axial disease, it is recommended to be cautious with the use of NSAIDs (age of patient, presence of comorbidity, etc.) (LE:1b / GR:A /LA: 92%).
6. In patients with axial disease, oral or intramuscular short-term low-dose glucocorticoids (4–12 weeks) could be considered for axial symptoms (LE:4 / GR:C /LA: 23%).

#### Evidence

In the studies selected for the SLR, the rate of PsA patients with axial disease was generally low (< 15%) [95]. Specific sub-analyses of axial manifestations were often lacking, although some positive evidence for tofacitinib [96] or ustekinumab [97] is available. Data from observational studies are also scarce [98]. Recently, a RCT, specifically designed to assess the efficacy and safety of biological treatment for the management of axial manifestations in patients with PsA, demonstrated that secukinumab 300 and 150 mg provided significant improvement in signs and symptoms of axial disease compared with placebo [99].

On the other hand, it has been published that axial disease in PsA patients seems to be different demographically, genetically, clinically and radiographically when compared with ankylosing spondylitis (AS) with or without psoriasis [100]. Axial PsA was associated with worse peripheral arthritis and less back pain [100]. However, more research is needed to confirm these findings.

Finally, it has also been suggested that axial inflammation in patients with PsA might respond better to corticosteroids than AS patients [101]. These are preliminary data that require further studies as well.

#### Expert´s comments and contributions

In daily practice, PsA can present with predominant axial disease but similarly with both, axial and peripheral manifestations. Furthermore, other manifestations such as enthesitis are quite common. That is why the experts encourage rheumatologists to properly evaluate the burden and impact of each manifestation on patient’s daily life to select the most appropriate treatment. But in general, in their experience, even in patients with both axial and peripheral disease, peripheral manifestations usually generate a greater impact or disability on PsA patients.

Experts also remark that sociodemographic and clinical characteristics of PsA patients are different than AS patients. Therefore, when considering non-steroidal anti-inflammatory drugs (NSAIDs) for axial symptoms in PsA, other factors like patient’s age or the presence of comorbidities should be considered [102]. If used, the experts also prefer short courses.

As exposed, we formulated a recommendation for the evaluation of short-term low-dose glucocorticoids for axial symptoms. However, it did not reach consensus probably due to insufficient evidence and the availability of alternative effective therapies for this domain.

### Enthesitis

#### Recommendations

7. In patients with enthesitis, a careful clinical history and physical examination should be performed to rule out other non-inflammatory conditions (plantar fasciitis, trochanteric bursitis, etc.), wide-spread pain syndromes or central sensitisation, especially in patients with multiple painful entheses (LE:2a / GR:B /DA: 88%).
8. In patients with enthesitis refractory to NSAIDs and/or local glucocorticoid injections, bDMARDs (except for abatacept), tofacitinib and apremilast are valid options in addition to reassess underlying inflammation with the use of imaging techniques such as ultrasound or MRI (LE:1b / DR:B /DA:92%).

#### Evidence

SRL reported a great variability in the enthesis evaluation of RCTs (physical examination, referred by the patient, imaging techniques, use of enthesitis indexes or counts, etc.).

Other SLRs and meta-analyses have depicted the efficacy of bDMARDs and tsDMARDs in PsA patients with enthesitis [5, 103, 104]. In in a recent meta-analysis, the estimated relative risks (RR) of enthesitis resolution in comparison to placebo across therapies were: RR = 1.99 (95% CI 1.36–2.90) for TNF inhibitors, RR = 2.31 (95% CI 1.60–3.34) for IL-17 inhibitors, RR = 1.41 (95% CI 1.02–1.95) for IL-12/23 inhibitors and RR = 0.85 (CI 0.74–0.99) for abatacept [103]. In a pooled analysis of tofacitinib, a higher rate of tofacitinib -treated patients achieved enthesitis resolution (Leed Enthesitis Index, LEI = 0) at month 3 compared with placebo. Further improvements in all enthesitis end-points were documented at month 6 [96]. Improvements have been reported with apremilast though resolution rates are modest [86, 87].

#### Expert´s comments and contributions

As in other sections, patient’s characteristics and impact of the disease should be a driver in the treatment decision-making.

When evaluating enthesitis, it is important to bear in mind the “enthesis organ”. It comprises the insertion of tendon and ligament to bone, but also adjacent tendon, bone fibrocartilage, fat pad, bursa, and synovium [105]. Therefore, clinical symptoms might be referred to any components of this complex and mechanical conditions, like fasciitis or bursitis, should be ruled out. Similarly, wide-spread pain syndromes or central sensitisation, especially in cases of multiple painful entheses [106], should be investigated. Finally, and based on available evidence, any bDMARD, tofacitinib or apremilast is a therapy to be considered in patients with enthesitis refractory to NSAIDs and/or local glucocorticoid injections.

### Dactylitis

#### Recommendations

9. In patients with polyarthritis, concomitant dactylitis should be treated like polyarthritis (LE:5 / GR:D / LA: 92%).
10. In patients with dactylitis as predominant manifestation, before the start of csDMARDs or a treatment change, a more conservative treatment (1 or 2 local glucocorticoids injections) might be considered depending on the number of dactylitis and their impact on patient (LE:3a / GR:C / LA: 88%).

#### Evidence

Regarding the csDMARDs, in our SLR, MTX at 12 weeks achieved complete resolution of dactylitis in 62.7% of patients [17]. In the case of bDMARDs, TNF inhibitors have shown to be significantly superior to placebo [103], but combined therapy with csDMARD was not statistically superior to TNF inhibitor monotherapy [44, 50]. Abatacept has depicted non-statistically but numerically more rate of complete resolution of dactylitis than placebo at 24 weeks (44.3 vs 34.0%) [60]. IL-17 inhibitors secukinumab and ixekizumab (IXE) achieved significant better results than placebo in several RCTs [66, 69, 71, 77]. However, in a recent RCT (SPIRIT H2H), there were no statistically significant differences between IXE and ADA at 24 weeks in the number of patients with complete resolution of dactylitis [72]. IL-12/23 inhibitor ustekinumab data indicate a significant decrease in the number of patients with dactylitis when compared to placebo [81, 82]. In pooled analysis of 2 RCT, the proportion of patients who achieved dactylitis resolution was statistically greater for tofacitinib vs. placebo at month 3 [96]. Apremilast has also demonstrated superiority when compared to placebo [87, 89]. Nash et al. in a pooled analysis showed a significant greater change in Dactylitis Severity Score (4.6 with tofacitinib 5 mg; and 5.8 with 10 mg versus 2.5 with placebo), and percent of patients achieving dactylitis resolution (43.3% with 5 mg and 55.2% with 10 mg versus 30.6% with placebo) at month 3. Both improvements were maintained to month 6 [96].

#### Expert´s comments and contributions

For the experts, it is important to emphasise that dactylitis is associated with radiographic changes in PsA [107] and, therefore, must be considered as a poor prognosis factor [4]. The presence of this manifestation should be seriously considered in the treatment decision-making.

Nonetheless, in patients with dactylitis as the predominant manifestation and low number of affected fingers and impact, 1 or 2 local glucocorticoids injections might be considered before csDMARDs [108].

### Skin and nail disease

11. In patients with PsA and significant skin involvement, if a bDMARDs is considered, IL-17 or IL-12/23 inhibitors may be preferred (LE:1b /GR:B /DA: 88%).

Skin involvement is one of the most evaluated domains in PsA RCTs. Most of them have used Psoriasis Area and Severity Index (PASI) 75 response, but PASI 100 response or total skin clearance is increasingly being assessed [50, 72, 76]. csDMARDs (mainly MTX), b (except for abatacept) and ts/DMARDs have shown superiority compared with placebo [5]. On the other hand, nail involvement was not frequently assessed among included patients, but when evaluated the Nail Psoriasis Severity Index (NAPSI) is the preferred score. The efficacy of b (except for abatacept) and ts/DMARDs has also been reported [5, 109].

#### Expert´s comments and contributions

Bearing in mind the results of SLRs and meta-analyses in moderate–severe psoriasis [110], the experts consider that in patients with PsA and significant skin involvement, IL-17 or IL-12/23 inhibitors may be preferred. In these cases, treatment decision-making should be shared with patient and dermatologist.

### Non-musculoskeletal manifestations and comorbidities

#### Recommendations

12. Comorbidities should be systematically evaluated and managed in all patients with PsA (LE:5 / DR:D / DA: 92%).

#### Evidence

It is mandatory to point out that many RCTs exclude patients with relevant comorbidities, limiting the evidence coming from these types of articles. This is a key point since frequent comorbidities in PsA patients may influence the rheumatologist’s selection of therapy [111].

Obesity is one of the most prevalent comorbid conditions. Different studies have observed that TNF inhibitors response is inferior in obese patients. So far, this finding has not been reported with other therapies in PsA [82, 112–114].

Regarding cardiovascular disease, evidence is still limited, but at least csDMARDs (especially MTX) and TNF inhibitors appear to be beneficial [109, 115, 116].

Smoking is another relevant risk habit/behaviour. The DANBIO registry showed that in PsA, smokers had a poorer response to TNF inhibitors compared to non-smokers. This was most pronounced in patients treated with infliximab or etanercept [117].

On the other hand, short-term clinical trials reported a somewhat elevated risk of depression in apremilast users, resulting in a label warning. However, long-term data and real-world evidence suggest that users of apremilast had similar rates of depression compared with users of other systemic non-corticosteroid PsA drugs [118, 119]. Similar considerations can be made for suicide.

Recently, a SLR and meta-analysis have concluded that bDMARDs and apremilast had a small effect on fatigue at 24 weeks in PsA RCTs and a higher effect on pain [120]. The OPAL Beyond and OPAL Broaden RCTs also described tofacitinib efficacy in PsA patients with fatigue [83, 121].

Treatment choices for patients with concurrent PsA and inflammatory bowel disease (IBD) should be made carefully. Therapies used to treat IBD may overlap with medications used to treat PsA. Common medications for IBD that have showed efficacy are MTX, TNF inhibitors, ustekinumab and specifically for ulcerative colitis, tofacitinib [122, 123].

Finally, for patients with PsA and uveitis, csDMARDs or TNF inhibitors (except etanercept) might be preferred [124, 125].

#### Expert´s comments and contributions

Comorbidities in PsA are extremely relevant as clinical evaluation, treatment response and adherence to treatment could be influenced by them. Thus, comorbidities should always be evaluated.

### Early PsA

#### Recommendations

13. In patients with PsA, an early and targeted treatment strategy like TICOPA is recommended (LE:1b / GR:B / DA: 88%).

#### Evidence

Few studies have specifically analysed the efficacy and safety of the selected drugs in patients with early PsA [91]. Inadequate response with MTX monotherapy in this sub-group of patients has been reported [20, 126], but a RCT and open-label extension recently published observed that around half of patients with early PsA achieved remission with initial combination treatment of MTX + TNF inhibitors that was maintained with MTX monotherapy afterwards [127].

On the other hand, The TIght COntrol of inflammation in early Psoriatic Arthritis (TICOPA) study was the first strategy trial in PsA to demonstrate that a treat-to-target strategy in early disease improves clinical outcomes over a 48-week period [128, 129]. In the TICOPA study, almost 40% of patients in the tight control, treat-to-target arm, were in minimal disease activity at 48 weeks, compared with 25% in the standard of care arm. Similar experiences have been reported in an open-label study [130].

#### Expert´s comments and contributions

As exposed, early diagnosis and treatment of PsA is associated with better outcomes. Thus, it is highly recommended to follow TICOPA recommendations and start treatment early in the course of the disease [129].

### Erosive PsA

#### Recommendations

14. In erosive PsA, early and tight treatment and monitoring is recommended (LE:1b / GR:B / DA: 96%).

#### Evidence

All therapies included in the SLR have shown efficacy in radiographic progression except for abatacept, and there are no data yet for apremilast or tofacitinib versus placebo [60, 131].

#### Expert´s comments and contributions

PsA is associated with structural damage as a result of bone and cartilage destruction. Risk factors for radiographic progression have been identified such as elevated CRP, number of tender and swollen joints, longer disease duration, and a high current damage index [132]. Thus, the experts consider that erosions presence is a poor prognostic factor in PsA and should prompt rheumatologist to an early and tight treatment and monitoring.

### Mono- vs combined therapy

#### Recommendations

15. In patients with PsA, the use of bDMARDs, or apremilast in monotherapy or in combination with csDMARDs, or tofacitinib in combination with csDMARDs, should be individualised (LE:5 / GR:D / LA: 100%).

#### Evidence

Robust evidence regarding the efficacy of combined therapy compared with monotherapy in PsA is lacking. It has been depicted that MTX increases bDMARDs survival and provides a slight reduction of immunogenicity [74, 133–135].

#### Expert´s comments and contributions

The expert’s perception is that combination therapy does not provide a robust difference in efficacy in relation to monotherapy. Although commonly no significant statistical differences can be detected when disease outcomes are analysed, a tendency to favour combination therapy is observed. However, the combination might also increase the risk of adverse events. Thus, every case should be evaluated individually**.**

### Risk management

#### Recommendations

16. Risk management recommendations for bDMARDs, tofacitinib and apremilast from regulatory agencies and scientific societies should be followed (L:1b / GR:B / LA:100%).

#### Evidence

Tofacitinib should be used with caution in patients with known risk factors for venous thromboembolism. Patients should also be reassessed periodically during treatment with tofacitinib to assess changes in the risk of venous thromboembolism [136].

Along with glucocorticoids, cs/b/tsDMARD therapy is associated with an increased risk of infections [137], which is one of the most frequently reported adverse events. More specifically, herpes zoster infections have been associated with the use of systemic glucocorticoids, tofacitinib and in a lesser extent with combined therapy with TNF inhibitors + csDMARDs [5, 138, 139]. There is little information regarding other therapies. The medical board of the National Psoriasis Foundation has recently recommended the recombinant zoster vaccine for all psoriasis and PsA patients > 50 years old and patients < 50 years old on tofacitinib, systemic glucocorticoids, or combination systemic treatment. Vaccination of patients < 50 years old on other systemic therapies may be considered on a case-by-case basis [138]. Similarly, data from IL-17 inhibitors (secukinumab and IXE) RCTs and observational studies suggest and increase risk of (mild) localised candidiasis [66, 69, 70, 72, 73].

On the other hand, an increased risk of injection site reactions (mostly mild) and cases of IBD have been described with IL-17 inhibitors (secukinumab and IXE) [72, 73, 140, 141].

Lastly, different registries like PSOLAR, ARTIS or DANBIO have provided evidence that neither csDMARDs nor TNF inhibitors or ustekinumab increase the overall malignancy risk (excluding non-melanoma skin cancer) [142]. Data from IL-17 inhibitors (pooled analysis) [141], tofacitinib and apremilast are similar so far [143].

#### Expert´s comments and contributions

All csDMARDs, bDMARDs, tofacitinib and apremilast present adverse events, some are common across therapies, and others might be class-related adverse events. Rheumatologists should monitor and treat all of them accordingly.

### Research agenda

The project also identified other issues and gaps that might be relevant for the decision-making, including pharmacogenetics data, biomarkers, basal IL levels, patient’s preferences or treatment stratification. Further research is needed to analyse their role in PsA and, therefore, in the treatment decision-making.

## Discussion

This project has generated a series of recommendations to treat PsA patients focused of non-resolved definitions of particular clinical phenotypes and that remains an uncertainty source and debate in the rheumatology community. These recommendations are based on the best evidence currently available, as well as the experience of an expert team and the subsequent evaluation of a broad group of rheumatologists with experience in the management of PsA.

Despite current therapeutic armamentarium and T2T strategy for PsA patients, there is still a great variability in the clinical practice regarding to the management of patients with PsA [144]. As a consequence, different national and international societies have published recommendations for the management of these patients in recent years [4, 10–13]. However, most of these recommendations are focused on a limited definition of the target populations and might not cover all clinical scenarios for which clinicians still seek guidance. Bearing in mind those limitations, we generated specific and practical recommendations to complete those formulated in previous documents [4, 10–13].

One of our main conclusions is that given the complexity and heterogeneity of multidomain PsA disease, we should carefully assess the impact of individual manifestations on patients (disability, quality of life, etc.). Along with objective data and prognostic profile, this viewpoint might help prioritise treatment selection and strategy in a shared decision-making.

We have discussed about patients with monoarthritis who might benefit from intra-articular glucocorticoids injections or short-term systemic low-dose glucocorticoids [92–94]. A rapid relief may be relevant especially considering the association of monoarthritis with disability and sick leave in PsA [91]. The same principle can be applied to enthesitis or dactilytis. Besides that, the experts would like to highlight that in daily practice it is vital to manage wide-spread pain syndromes or central sensitisation, especially in patients with multiple painful entheses as these syndromes are quite frequent in PsA [106] and might lead to false diagnosis of therapeutic failure or even overtreatment. In line with current evidence (basic science and clinical data), in PsA patients with several non-musculoskeletal manifestation like uveitis, IBD or skin/nail disease, specific therapies might be preferred [110, 122–125]. The presence of certain comorbidities might negatively interfere or, the opposite, might prompt physician to select specific therapies [82, 109, 111–117]; thus, it should be systematically evaluated and considered in the treatment selection. Anti-IL-23 drugs (guselkumab, tildrakizumab) [145] are likely to eventually gain a place as a therapeutic target, when experience is gained in different profiles and efficacy and safety are proven in real life. Finally, we also encourage physicians to follow T2T strategies in early PsA as they are providing promising results [128–130].

On the other hand, the main limitation of this work is the lack of published quality evidence that specifically address some of the open questions on the selected clinical phenotypes. For this reason, expert opinion is the only way to deliver recommendations that try to resolve uncertainty. In this regard, a strength of the study is the broad evaluation of the set recommendations that was extended to a significant number of rheumatologists through a Delphi process. A very high level of agreement in all but one recommendation was reached, which increases the validity of the recommendations.

In conclusion, treatment decisions are not always straightforward in PsA. We are confident that these recommendations will find their way into the clinic for a better care of the PsA patients in the real-world setting.

## Supplementary Information

Below is the link to the electronic supplementary material.

Supplementary file1 (DOCX 19 KB)
