# Supplementary material for: Management of particular clinical situations in psoriatic arthritis: an expert’s recommendation document based on systematic literature review and extended Delphi process
Source: Rheumatol Int. 2021 May 2;41(9):1549–65. doi: 10.1007/s00296-021-04877-5 (PMC8316175; doi:10.1007/s00296-021-04877-5)
Supplement: Supplementary file 1 — Supplementary file1 (DOCX 19 KB) [file 296_2021_4877_MOESM1_ESM.docx]

Search strategies and retrieved articles.

**Table 1.** . Search strategy in Pubmed.

| **#** | **Search terms** | **References** |
| --- | --- | --- |
| 1 | ("Arthritis, Psoriatic"[Mesh] OR arthritic psoriasis[tw] OR psoriatic arthritis[tw]) AND (Adalimumab[tw] OR Humira[tw] OR Amjevita[tw] OR Hulio[tw] OR Cyltezo[tw] OR Alefacept[tw] OR Amevive[tw] OR Auranofin[tw] OR Ridaura[tw] OR Certolizumab Pegol[tw] OR Certolizumab[tw] OR Cimzia[tw] OR Chlorambucil[tw] OR Chloraminophene[tw] OR Chlorbutin[tw] OR Leukeran[tw] OR lympholysin[tw] OR Amboclorin[tw] OR Gold Sodium Thiomalate[tw] OR Myochrysine[tw] OR Myochrysin[tw] OR Myocrisin[tw] OR Tauredon[tw] OR Aurothiomalate[tw] OR Miocrin[tw] OR Thiomalatoaurate[tw] OR Thiomalate[tw] OR "Gold Thiomalic Acid"[tw] OR miocrisina[tw] OR Golimumab[tw] OR Simponi[tw] OR Hydroxychloroquine[tw] OR Hydroxychlorochin[tw] OR Hydroxychloroquin[tw] OR Oxychloroquine[tw] OR Oxychlorochin[tw] OR Oxychloroquin[tw] OR Plaquenil[tw] OR Dolquine[tw] OR hidroxicloroquina[tw] OR Xanban[tw] OR Infliximab[tw] OR Renflexis[tw] OR Inflectra[tw] OR Remicade[tw] OR Flixabi[tw] OR Remsina[tw] OR Zessly[tw] OR Ixekizumab[tw] OR Taltz[tw] OR Leflunomide[tw] OR Arava[tw] OR Lefluartil[tw] OR Leflunomida[tw] OR Methotrexate[tw] OR Amethopterin[tw] OR Mexate[tw] OR Reumatrex[tw] OR Trexall[tw] OR Otrexup[tw] OR Rasuvo[tw] OR cosentyx[tw] OR secukinumab[tw] OR Tofacitinib[tw] OR Xeljanz[tw] OR tasocitinib[tw] OR Otezla[tw] OR apremilast[tw] OR apremilast[tw] OR Otezla[tw] OR Azathioprine[tw] OR Azothioprine[tw] OR Imurel[tw] OR Immuran[tw] OR imuran[tw] OR Chloroquine[tw] OR Chlorochin[tw] OR Chingamin[tw] OR Chloroquin[tw] OR Khingamin[tw] OR Nivaquine[tw] OR Aralen[tw] OR Arequin[tw] OR Arechine[tw] OR Resochin[tw] OR Cyclophosphamide[tw] OR Sendoxan[tw] OR Cytophosphane[tw] OR Cytoxan[tw] OR Endoxan[tw] OR Neosar[tw] OR Procytox[tw] OR Cyclophosphane[tw] OR Genoxal[tw] OR Cyclosporine[tw] OR Cyclosporin[tw] OR CIclosporin[tw] OR Neoral[tw] OR Sandimmun[tw] OR Ciqorin[tw] OR Gengraf[tw] OR Cyclosporine[tw] OR Cyclosporin[tw] OR Neoral[tw] OR Sandimmun[tw] OR Ciqorin[tw] OR Gengraf[tw] OR Etanercept[tw] OR Enbrel[tw] OR Erelzi[tw] OR Benepali[tw] OR Mycophenolic Acid[tw] OR mycophenolate mofetil[tw] OR CellCept[tw] OR myfortic[tw] OR Sulfasalazine[tw] OR Salicylazosulfapyridine[tw] OR Azulfidine[tw] OR Salazopyrin[tw] OR Salazopyrina[tw] OR Sulphasalazine[tw] OR Asulfidine[tw] OR pleon[tw] OR “pyralin”[tw] OR sulfasalazine[tw] OR ucine[tw] OR ustekinumab[tw] OR stelara[tw]) | 334 |

**Table 2.** Search strategy in Embase.

| **#** | **Search terms** | **References** |
| --- | --- | --- |
| **1** | ('psoriatic arthritis'/exp OR 'psoriatic arthritis' OR 'arthritic psoriasis') AND  (((((('adalimumab'/exp OR adalimumab OR 'humira'/exp OR humira OR 'amjevita'/exp OR amjevita OR 'hulio'/exp OR hulio OR 'cyltezo'/exp OR cyltezo OR 'alefacept'/exp OR alefacept OR 'amevive'/exp OR amevive OR 'auranofin'/exp OR auranofin OR 'ridaura'/exp OR ridaura OR 'certolizumab'/exp OR certolizumab) AND pegol OR 'certolizumab'/exp OR certolizumab OR 'cimzia'/exp OR cimzia OR 'chlorambucil'/exp OR chlorambucil OR 'chloraminophene'/exp OR chloraminophene OR 'chlorbutin'/exp OR chlorbutin OR 'leukeran'/exp OR leukeran OR 'lympholysin'/exp OR lympholysin OR 'amboclorin'/exp OR amboclorin OR 'gold'/exp OR gold) AND ('sodium'/exp OR sodium) AND ('thiomalate'/exp OR thiomalate) OR 'myochrysine'/exp OR myochrysine OR 'myochrysin'/exp OR myochrysin OR 'myocrisin'/exp OR myocrisin OR 'tauredon'/exp OR tauredon OR 'aurothiomalate'/exp OR aurothiomalate OR 'miocrin'/exp OR miocrin OR thiomalatoaurate OR 'thiomalate'/exp OR thiomalate OR 'gold'/exp OR gold) AND thiomalic AND ('acid'/exp OR acid) OR miocrisina OR 'golimumab'/exp OR golimumab OR 'simponi'/exp OR simponi OR 'hydroxychloroquine'/exp OR hydroxychloroquine OR hydroxychlorochin OR hydroxychloroquin OR 'oxychloroquine'/exp OR oxychloroquine OR oxychlorochin OR 'plaquenil'/exp OR plaquenil OR dolquine OR hidroxicloroquina OR xanban OR 'infliximab'/exp OR infliximab OR 'renflexis'/exp OR renflexis OR 'inflectra'/exp OR inflectra OR 'remicade'/exp OR remicade OR 'flixabi'/exp OR flixabi OR remsina OR 'zessly'/exp OR zessly OR 'ixekizumab'/exp OR ixekizumab OR 'taltz'/exp OR taltz OR 'leflunomide'/exp OR leflunomide OR 'arava'/exp OR arava OR lefluartil OR leflunomida OR 'methotrexate'/exp OR methotrexate OR 'amethopterin'/exp OR amethopterin OR 'mexate'/exp OR mexate OR 'reumatrex'/exp OR reumatrex OR 'trexall'/exp OR trexall OR 'otrexup'/exp OR otrexup OR 'rasuvo'/exp OR rasuvo OR 'cosentyx'/exp OR cosentyx OR 'secukinumab'/exp OR secukinumab OR 'tofacitinib'/exp OR tofacitinib OR 'xeljanz'/exp OR xeljanz OR 'tasocitinib'/exp OR tasocitinib OR 'apremilast'/exp OR apremilast OR 'otezla'/exp OR otezla OR 'azathioprine'/exp OR azathioprine OR 'azothioprine'/exp OR azothioprine OR 5 'imurel'/exp OR imurel OR 'immuran'/exp OR immuran OR 'imuran'/exp OR imuran OR 'chloroquine'/exp OR chloroquine OR 'chlorochin'/exp OR chlorochin OR chingamin OR 'chloroquin'/exp OR chloroquin OR khingamin OR 'nivaquine'/exp OR nivaquine OR 'aralen'/exp OR aralen OR arequin OR 'arechine'/exp OR arechine OR 'resochin'/exp OR resochin OR 'cyclophosphamide'/exp OR cyclophosphamide OR 'sendoxan'/exp OR sendoxan OR 'cytophosphane'/exp OR cytophosphane OR 'cytoxan'/exp OR cytoxan OR 'endoxan'/exp OR endoxan OR 'neosar'/exp OR neosar OR 'procytox'/exp OR procytox OR 'cyclophosphane'/exp OR cyclophosphane OR 'genoxal'/exp OR genoxal OR 'ciclosporin'/exp OR ciclosporin OR 'cyclosporine'/exp OR cyclosporine OR 'cyclosporin'/exp OR cyclosporine OR 'neoral'/exp OR neoral OR 'sandimmun'/exp OR sandimmun OR ciqorin OR 'gengraf'/exp OR gengraf OR 'etanercept'/exp OR etanercept OR 'enbrel'/exp OR enbrel OR 'erelzi'/exp OR erelzi OR 'benepali'/exp OR benepali OR mycophenolic) AND ('acid'/exp OR acid) OR 'mycophenolate'/exp OR mycophenolate) AND mofetil OR 'cellcept'/exp OR cellcept OR 'myfortic'/exp OR myfortic OR 'salicylazosulfapyridine'/exp OR salicylazosulfapyridine OR 'azulfidine'/exp OR azulfidine OR 'salazopyrin'/exp OR salazopyrin OR 'salazopyrina'/exp OR salazopyrina OR 'sulphasalazine'/exp OR sulphasalazine OR asulfidine OR pleon OR pyralin OR 'sulfasalazine'/exp OR sulfasalazine OR ucine OR 'ustekinumab'/exp OR ustekinumab OR 'stelara'/exp OR stelara) AND ('Article'/it OR 'Article in Press'/it OR 'Review'/it) | 31 |

**Table 3.** Search strategy in the Cochrane Library.

| **#** | **Search terms** | **References** |
| --- | --- | --- |
| **1** | MeSH descriptor: [Arthritis, Psoriatic] explode all trees | 411 |
| **2** | (Adalimumab OR Humira OR Amjevita OR Hulio OR Cyltezo OR Alefacept OR  Amevive OR Auranofin OR Ridaura OR Certolizumab Pegol OR Certolizumab OR Cimzia OR Chlorambucil OR Chloraminophene OR Chlorbutin OR Leukeran OR lympholysin OR Amboclorin OR Gold Sodium Thiomalate OR Myochrysine OR Myochrysin OR Myocrisin OR Tauredon OR Aurothiomalate OR Miocrin OR Thiomalatoaurate OR Thiomalate OR Gold Thiomalic Acid OR miocrisina OR Golimumab OR Simponi OR Hydroxychloroquine OR Hydroxychlorochin OR Hydroxychloroquin OR Oxychloroquine OR Oxychlorochin OR Oxychloroquin OR Plaquenil OR Dolquine OR hidroxicloroquina OR Xanban OR Infliximab OR Renflexis OR Inflectra OR Remicade OR Flixabi OR Remsina OR Zessly OR Ixekizumab OR Taltz OR Leflunomide OR Arava OR Lefluartil OR Leflunomida OR Methotrexate OR Amethopterin OR Mexate OR Reumatrex OR Trexall OR Otrexup OR Rasuvo OR cosentyx OR secukinumab OR Tofacitinib OR Xeljanz OR tasocitinib OR Otezla OR apremilast OR apremilast OR Otezla OR Azathioprine OR Azothioprine OR Imurel OR Immuran OR Imuran OR Chloroquine OR Chlorochin OR Chingamin OR Chloroquin OR Khingamin OR Nivaquine OR Aralen OR Arequin OR Arechine OR Resochin OR Cyclophosphamide OR Sendoxan OR Cytophosphane OR Cytoxan OR Endoxan OR Neosar OR Procytox OR Cyclophosphane OR Genoxal OR Cyclosporine OR Cyclosporin OR CIclosporin OR Neoral OR Sandimmun OR Ciqorin OR Gengraf OR Cyclosporine OR Cyclosporin OR Neoral OR Sandimmun OR Ciqorin OR Gengraf OR Etanercept OR Enbrel OR Erelzi OR Benepali OR Mycophenolic Acid OR mycophenolate mofetil OR CellCept OR myfortic OR Sulfasalazine OR Salicylazosulfapyridine OR Azulfidine OR Salazopyrin OR Salazopyrina OR Sulphasalazine OR Asulfidine OR pleon OR pyralin OR sulfasalazine OR ucine OR ustekinumab OR stelara):ti,ab,kw (Word variations have been searched) | 39.858 |
| **3** | #1 AND #2 in Trials | 272 |
| **4** | #1 AND #2 in Cochrane Reviews, Cochrane Protocols | 4 |
